# Outcomes of One-Stage Surgical Repair for Berry Syndrome in Neonates

**DOI:** 10.3389/fcvm.2021.790303

**Published:** 2022-01-26

**Authors:** Xu-Cong Shi, Jian-Bin Weng, Jin Yu, Xiao-Hui Ma, Yi-Qing Pu, Li-Yang Ying, Jian-Gen Yu

**Affiliations:** ^1^Department of Cardiac Surgery, The Children's Hospital, Zhejiang University School of Medicine, National Clinical Research Center for Child Health, Hangzhou, China; ^2^Department of Neurosurgery, The Children's Hospital, Zhejiang University School of Medicine, National Clinical Research Center for Child Health, Hangzhou, China; ^3^Department of Ultrasound Diagnosis, The Children's Hospital, Zhejiang University School of Medicine, National Clinical Research Center for Child Health, Hangzhou, China; ^4^Department of Radiology, The Children's Hospital, Zhejiang University School of Medicine, National Clinical Research Center for Child Health, Hangzhou, China; ^5^Department of Operation Room, The Children's Hospital, Zhejiang University School of Medicine, National Clinical Research Center for Child Health, Hangzhou, China

**Keywords:** berry syndrome, neonate, one-stage surgical repair, outcomes, right pulmonary stenosis

## Abstract

**Background:**

Berry syndrome is a challenging disease for surgeons to make early diagnosis and successful surgical correction in the neonatal period. Here, we summarized the clinical features of three neonates with berry syndrome in our center to optimize the therapeutic effect in the future.

**Methods:**

From January 2014 to December 2019, three neonates with berry syndrome underwent one-stage surgical repair in our center. We mainly used two different surgical techniques to repair it, and collected clinical data retrospectively from hospitalization history, outpatient records, and telephone follow-up.

**Results:**

The age at operation was 28, 8, and 8 days and the body weight was 3.65, 3.86, and 3.0 kg, respectively. The morphology of the interrupted aortic arch (IAA) was type A in two patients and type B in one patient. The aortopulmonary window (APW) morphology was type IIa, III, and IIb, respectively. The phenotype of the IAA type B combined with APW type III in our second patient was reported for the first time so far. All patients survived and were followed up to date. The second patient using intra-aortic baffle experienced twice reoperation for right pulmonary artery (RPA) stenosis. All patients grew well so far.

**Conclusion:**

Once diagnosed in the neonatal period, berry syndrome can be safely corrected by one-stage surgical repair in experienced cardiac centers. Considering the variability of the location where the RPA arises from the posterior wall of the aorta, it is difficult to find the best surgical method for each patient.

## Introduction

Berry syndrome is a rare disease and manifests complex cardiac anomalies involving interrupted aortic arch (IAA) or hypoplastic aortic arch (HAA) or coarctation of the aorta (CoA), aortopulmonary window (APW), the aortic origin of the right pulmonary artery (AORPA), and intact ventricular septum (1). The incidence of this syndrome among patients with congenital heart diseases is 0.046%, according to literature reports (2). Timely treatment after birth is very important, because most of the patients die soon after birth while the surviving patients will develop pulmonary hypertension quickly. This complex deformity has been reported in about 100 patients in the English literature since 1982 Berry first described (1). For unoperated patients, the mortality rate was 100% and the median age at death was 1 month (1). Only a small part of patients (31 cases) was operated at the neonatal period with 6 neonates (6/31, 20%) who died post-operatively (1). It is a great challenge for surgeons to make early diagnosis and successful surgical correction in the neonatal period and to make sure of the long-term survival of the patients.

Herein, we collected the clinical information of three neonates with berry syndrome that successfully underwent one-stage surgical repair at The Children's Hospital, Zhejiang University School of Medicine, since January 2014 through December 2019. Luckily, all the patients were survival and followed up for 2–6 years. Echocardiography was performed regularly to check the development of descending aortic arch and bilateral pulmonary arteries. We present here our experience of one-stage repair to optimize the therapeutic effect of this rare complex deformity in the future.

## Patients and Methods

From January 2014 to December 2019, three neonates diagnosed as berry syndrome were admitted to our center and underwent one-stage complete repair. The hospital ethics committee approved this study and waived the need for individual consent. We collected clinical data of all patients retrospectively from hospitalization history, outpatient records, and telephone follow-up.

### Pre-Operative Characteristics

**Figure 1:**
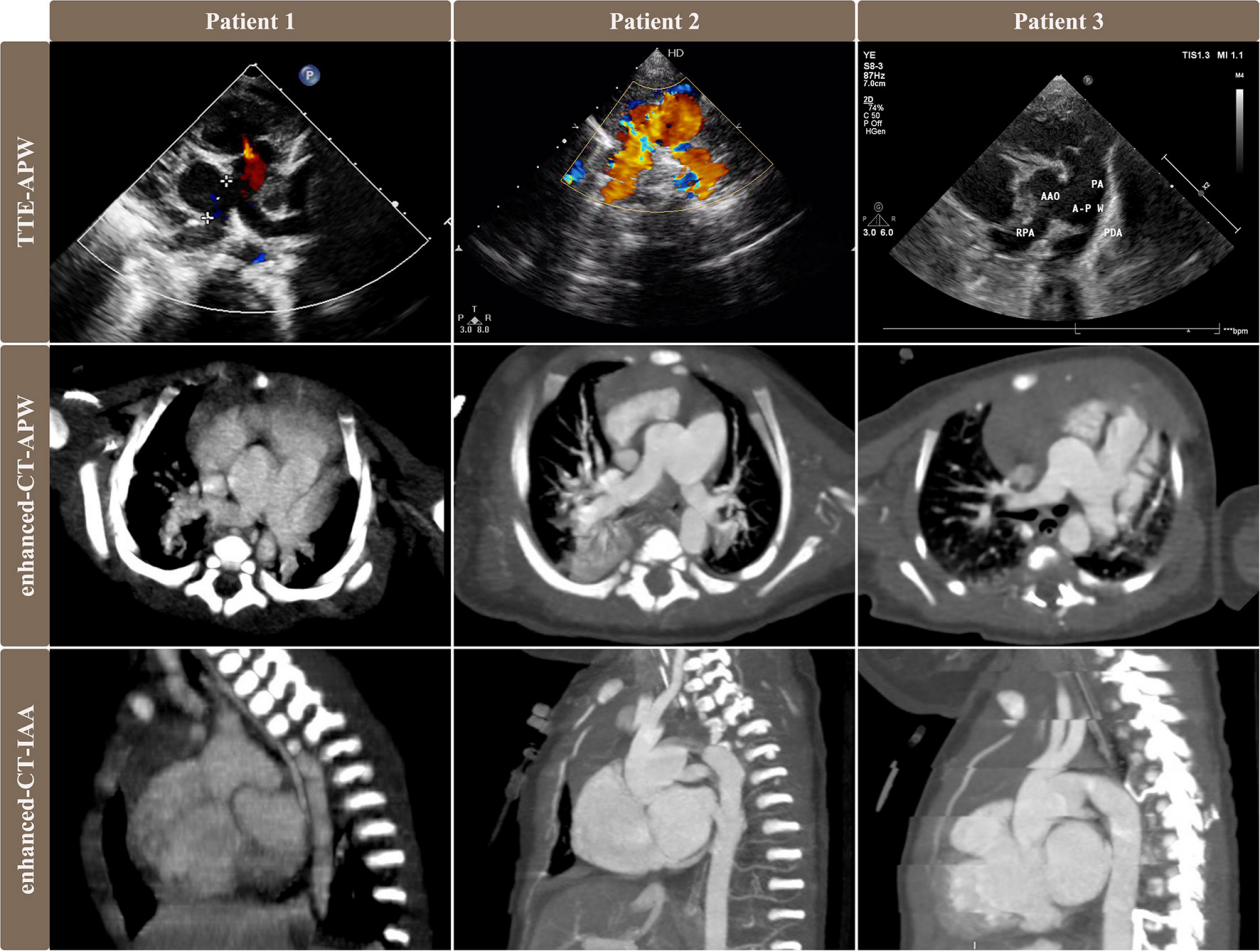
Pre-operative images of transthoracic echocardiography and contrast-enhanced CT scan showed different types of APW, AORPA, and IAA in 3 patients. Images of the first and second rows showed the relationship among RPA, AAO, APW, and PA. Patient No. 1 was APW type IIa, patient No. 2 was APW type III, and patient No. 3 was APW type IIb. Images of the third rows were sagittal views of CT exhibiting IAA that descending aorta originated from PDA.TTE, transthoracic echocardiography; APW, aortopulmonary window; AORPA, the aortic origin of the right pulmonary artery; IAA, interrupted aortic arch; RPA, right pulmonary artery; AAO, ascending aorta; PA, pulmonary artery; PDA, patent ductus arteriosus.

Clinical symptoms include cyanosis, tachypnea/dyspnea/respiratory infection, cardiac murmur, chronic heart failure/poor peripheral perfusion, and poor feeding/anuria. Berry syndrome was diagnosed by transthoracic echocardiography and cardiac contrast-enhanced CT scan in all patients (Figure 1). The IAA morphology was specified according to the classification reported by Celoria and Patton (3). The APW type was based on the original classification of Mori (4) and Berry's supplementary pathologic findings (5).

All three patients were female, full-term, and monotocous newborns from vaginal delivery. Patient No. 1 and 3 were IAA type A and had a stable condition that only air inhalation was enough before operation under the fundamental diuretic treatment. Patient No. 1 was hospitalized due to cough and jaundice with signs of cyanosis and hepatomegaly, diagnosed as pneumonia and treated with antibiotics. Patient No. 3 was admitted to the hospital for prenatal diagnosis of cardiac malformations. Patient No. 2 was IAA type B and developed severe dyspnea and circulatory failure shortly after birth, requiring endotracheal intubation with mechanical ventilation. This patient received prostaglandin E1 infusion (9 ng/kg∙min) and depended on mechanical ventilation before surgery. All three patients received one-stage surgical repair and survived with being followed up to date.

### Operative Technique

All patients were operated soon after clinical diagnosis. Median sternotomy was routinely required. Aorta and its branches, and left and right pulmonary arteries (RPA) were widely mobilized. Cardiopulmonary bypass (CPB) was performed using deep hypothermia with low-flow antegrade selective cerebral perfusion (DHLF) during aortic arch repair, the same as simple IAA repair operation. Briefly, two arterial cannulas were connected through a Y-connector. One 8F arterial cannula was inserted into the ascending aorta. Another 8F arterial cannula was inserted into the anterior wall of ductus arteriosus and then arrived to the descending aorta. The superior and inferior vena cava cannulated conventionally by two 16F venous cannulas separately. Before the start of CPB, left and RPA were blocked. The aorta was cross-clamped and cold blood cardioplegia was infused into the aortic root, cooling the patient to the appropriate body temperature at 20. Then remove the cannula in ductus arteriosus and adjust the ascending aortic cannula into the innominate artery to initiate DHLF. Ductus arteriosus was first isolated distally and ligated, then all the ductal tissue was resected. For minimizing the tension, we should mobilize the aortic arch and descending aorta as far as possible. Then the descending aorta was anastomosed to the aortic arch by an end-to-side anastomosis with a bovine pericardial patch augmentation at the anterior wall. Completing the repair of the aortic arch, we can restore the aortic cannula to the normal arch position. Without time-bound, we could examine carefully and thoroughly in each patient to confirm the type of APW, origins of the coronary arteries, and the opening of the right pulmonary artery (RPA) and left pulmonary artery (LPA), to make a better operation plan.

**Figure 2:**
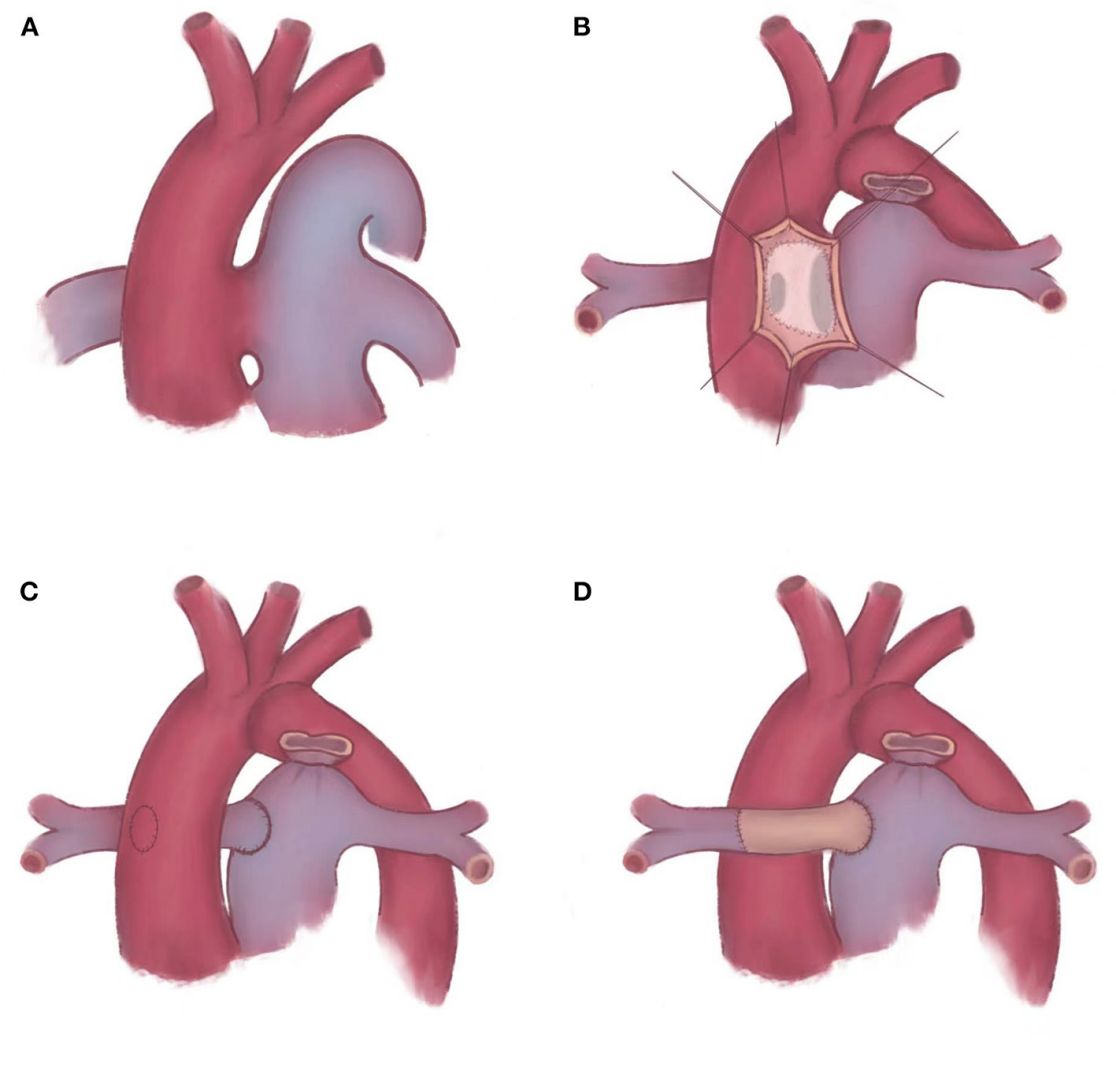
Schematic diagram of repairing APW and AORPA. **(A)** Berry syndrome is a complex deformity, such as APW, AORPA, and IAA. **(B)** An trimmed intra-aortic baffle patch is placed to reconstruct the continuity of RPA. **(C,D)** AORPA is cut-off directly and reconstructed to the right lateral side of MPA. **(C)** RPA is anastomosed to the MPA *in situ*. **(D)** RPA is anastomosed to the MPA at the anterior of the aorta with patch augmentation in a similar fashion to the LeCompte maneuver. APW, aortopulmonary window; AORPA, the aortic origin of the right pulmonary artery; IAA, interrupted aortic arch; RPA, right pulmonary artery; MPA, main pulmonary artery.

We chose different surgical methods in different patients according to the type of cardiac malformation (Figure 2A). In two patients who were IAA type A, patient No. 1 was APW type IIa [accompanied with a straddling RPA, whose blood flow comes from the ascending aorta (AAO) but tissue maintains the continuity with the main pulmonary artery (MPA) and LPA (6)] and patient No. 3 was APW type IIb [accompanied with a completely separated RPA, whose blood flow and tissue were both from the AAO (6)], we applied RPA cut-off and replantation technique. Cut the RPA directly from the posterior wall of the aorta. Separate MPA along the vertical incision in the APW. Isolate the RPA and MPA as much as possible to ensure sufficient length to connect. The RPA was then directly reconstructed to the right lateral side of the MPA in an end-to-side anastomosis in patient No. 1 (Figure 2C). In patient No. 3, RPA was anastomosed to the MPA at the anterior of the aorta with patch augmentation in a similar fashion to the LeCompte maneuver (Figure 2D). In patient No. 2 with IAA type B and APW type III, a trimmed intra-aortic baffle patch was used to close the APW and construct the connection from RPA to MPA simultaneously, redirecting the blood flow from the MPA to the RPA (Figure 2B). We used an autologous anterior wall of the pulmonary artery (PA) as the intra-aortic baffle patch.

### Follow-Up

Patients were followed at 1, 3, 6, 12, 18, 24, and every 12 months after discharge. Physical examination, echocardiography, electrocardiography, and cardiac function were evaluated in our outpatient clinics.

## Results

For patients No. 1, 2, and 3, the age at operation was 28, 8, and 8 days and the body weight was 3.65, 3.86, and 3.05 kg, respectively. The IAA morphology was type A in two patients (66.7%) and type B in one patient (33.3%). The APW morphology was type II in two patients, type III in one patient. The CPB time was 133, 155, and 192 min and aortic cross-clamp (ACC) time was 94, 101, and 108 min, respectively. The time for DHLF was 50, 79, and 40 min, respectively. Patient No. 1 was treated with delayed chest closure and Treprostinil due to cardiac enlargement, and her chest was closed successfully on the second day after surgery. Patient No. 2 required defibrillation at the time of post-operative cardiac resuscitation. The time for post-operative mechanical ventilation was 3, 4, and 2 days, and the time in the intensive care unit was 10, 21, and 9 days, respectively. Patient No. 2 experienced massive gastrointestinal hemorrhage and shock on the 6th day after the operation. After active treatment, the patient was cured, but the retention time of ICU was significantly prolonged.

**Figure 3:**
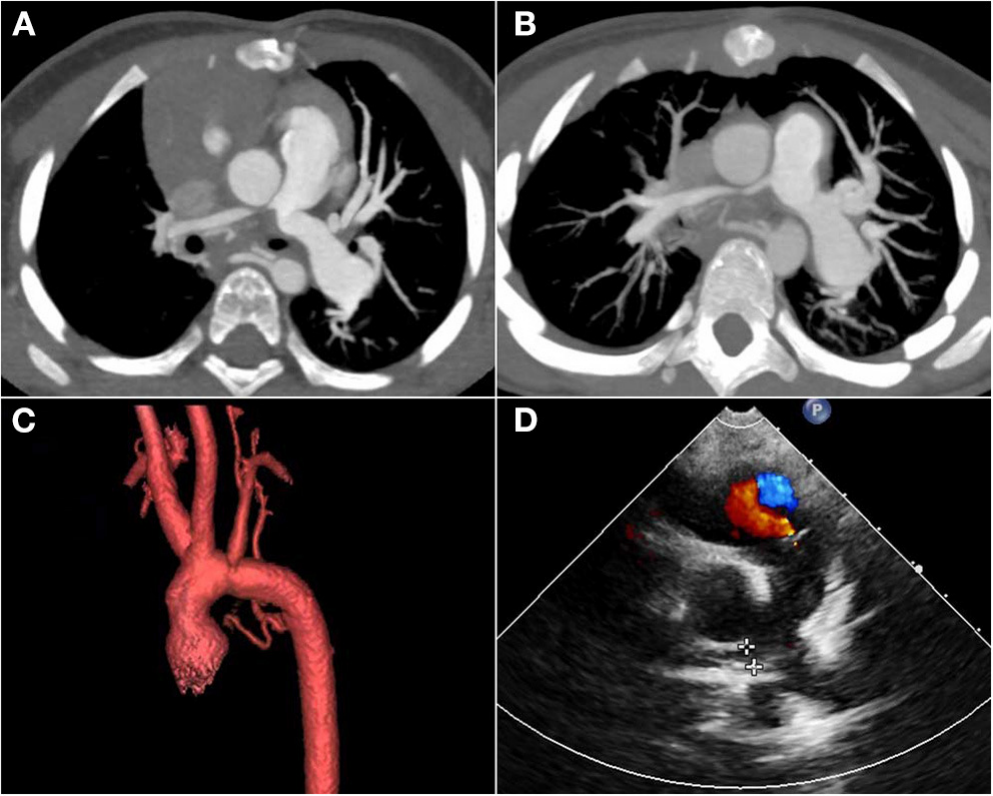
Post-operative follow-up images of patient No. 2. **(A)** CT images showed that ascending aorta oppressed the opening of RPA and LPA 12 months after the primary operation. **(B)** CT images showed RPA stenosis again 6 months after the first re-operation. **(C)** The post-operative three-dimensional imaging of the aortic arch showed that the aortic arch and its branches were in good shape. **(D)** The latest ultrasound images showed good development of RPA with a gradient of 9 mmHg. RPA, right pulmonary artery; LPA, left pulmonary artery.

Reoperations were occurred in patient No. 2 two times during the follow-up period with a rate of 33.3%. She underwent surgical patch angioplasty at the 12th and 22nd months after the primary operation due to RPA stenosis. Regular ultrasound screening after operation found the flow velocity of RPA increased, then CT confirmed both RPA and LPA stenosis (Figure 3A). Therefore, this patient underwent the first reoperation at the 12th month after the primary operation. During the operation, we severed the aorta above the aortic root, and then fully dissociated MPA, RPA, and LPA. After incision of MPA, we found that the opening of LPA was slightly narrowed and the opening of RPA was nearly closed. Then the stenosis segment of RPA was resected, and the posterior wall of RPA was anastomosed to MPA directly and the anterior wall of RPA was expanded with autologous pericardium. Finally, the aorta was anastomosed *in situ*. However, this patient was very unlucky. At the follow-up of 6 months after the first reoperation, RPA stenosis was found again (Figure 3B) and was re-operated at the 22nd month after the primary operation. According to the latest follow-up, both the RPA and LPA are well-developed (Figure 3D), and the child shows normal growing and development as expected with a height of 121 cm and body weight of 20 kg at the age of nearly 6 years old.

Follow-up time was 6, 5, and 2 years, respectively. No feeding difficulties and left vocal cord paralysis occurred in any patients. No patients occurred supra-valvar aortic stenosis or aortic arch stenosis, with a flow velocity of the reconstructed aorta at 1.1, 1.5, 1.2 M/S, respectively (Figure 3C). At the latest follow-up, all three patients had good cardiac function (New York Heart Association functional class I). The left ventricular ejection fraction was 68, 62, and 64% and the RPA gradient was 25, 9, and 23 mmHg, respectively. Tricuspid regurgitation was trivial in all patients. Clinical data were summarized in Table 1.

**Table 1:** Characteristics of patients.

**Parameter**	**Patient 1**	**Patient 2**	**Patient 3**
Age, d	28	8	8
Weight, kg	3.65	3.86	3.05
IAA type	A	B	A
APW type	IIa	III	IIb
APW size, cm	1.1	1.5	1.2
Repair method^*^	2	1	2
CPB, min	133	155	192
ACC, min	94	101	108
DHLF, min	50	79	40
ICU, d	10	21	9
Ventilator, d	3	4	2
Follow-up, y	6	5	2
LV-EF, %,	68	62	64
DAO, M/S	1.0	1.6	1.3
RPA gradient	25	9	23

IAA, interrupted aortic arch; APW, aortopulmonary window; RPA, right pulmonary artery; CPB, cardiopulmonary bypass; ACC, aorta cross-clamp; DHLF, deep hypothermia with low-flow antegrade selective cerebral perfusion; ICU, Intensive care unit; DAO, descending aorta. Repair method^*^, 1 was intra-aortic baffle method, 2 was RPA cut-off and replantation technique.

## Discussion

Berry syndrome is a rare and life-threatening cardiac malformation, whose correct diagnosis and treatment in the neonatal period are challenging both to the cardiac sonographer and the cardiac surgeon. Berry et al. (5) described the possible embryogenesis that APW might affect the formation of AORPA, then the blood flow through the aortic arch would be reduced, leading to HAA/CoA/IAA. Due to the complexity, so far it is still difficult to make a precise diagnosis and one-stage surgical correction in the neonatal period.

The anatomic focus mainly concentrates on the type and range of IAA, and the location and size of APW and AORPA. The perfusion of the lower body is entirely supplied by patent ductus arteriosus (PDA), and the oxygenated blood flow depends on the APW between the aorta and pulmonary arteries. Severe pulmonary hypertension appears early after birth due to massive pulmonary blood flow. Thus, Hu et al. (6) recommended early surgical repair even in the neonatal period to avoid irreversible hypertension of the pulmonary vascular bed and recover the normal blood circulation to the organs of the lower body. In our center, all three cases were neonates, and two patients with A type of IAA were relatively stable before the operation, requiring nor PGE 1 or mechanical ventilation, but one patient with B type of IAA manifested renal failure and low output syndrome when admitted to hospital. Anatomically, IAA type B occurs between the left common carotid artery and the left subclavian artery (7); therefore, the blood flow passing through the PDA to the systemic circulation is more in IAA type B than that in IAA type A. When PDA shrinks naturally after birth, left ventricular dysfunction, circulatory collapse, renal insufficiency, and metabolic acidosis may occur due to the increase of left ventricle afterload and hypoperfusion of the lower body. Thus, IAA type B in this syndrome shows a greater influence on systemic circulation and demands for earlier diagnosis and intervention.

There are two kinds of surgical strategies. One strategy is a one-stage repair that all cardiac malformations were corrected in one operation, including closed of APW, reconstruction of the RPA, and repair of the aortic arch. The other one is the two-stage repair that involves pulmonary arterial banding at the first stage and then complete repair after the ventilation status improves. The researchers in most of the reported cases advocate one-stage surgical repair. However, two-stage repair should be considered in premature infants or small-for-gestational-age infants due to lower risk (8). Ando et al. (9) presented a case of rapid two-stage repair that a 14-day-old girl weighing 3.3 kg underwent bilateral pulmonary arterial banding and complete repair. They thought a rapid two-stage approach could reduce the risk of complete repair. While, according to the analysis of Bi et al. (1), there was no difference between one-stage repair and two-stage repair in mortality or survival time. Combined with the risk and difficulty of operation, we still recommend one-stage surgical correction in the mature cardiac centers.

So far, it is not often seen that such patients can be clearly diagnosed and treated with one-stage surgical correction in the neonatal period. Three cases within 6 years are valuable experience. In our center, we used selective cerebral perfusion for IAA. Repair of the aortic arch was achieved by using artificial material (Bovine pericardial patch or Gore-Tex patch) and end-to-lateral anastomosis of posterior wall of aorta offering the potential vascular growth. As for APW and AORPA, an autologous patch (pericardial patch or aorto-pulmonary wall) was needed, while circumferential suture lines on all the anastomoses should be avoided. We found the total operative risk of this syndrome may be similar to that of simple IAA with a ventricular septal defect. Immediate and mid-term hemodynamics and clinical outcomes seem to be satisfactory. During the follow-up period up to 5–6 years, the one-stage surgical repair of berry syndrome in our center shows an excellent survival rate, and there was no evidence of persistent pulmonary hypertension. Echo result showed no residual APW, no residual obstruction in the reconstructed aortic arch, and good LV function with EF of 65%.

Repair of IAA and APW is a conventional procedure in many centers, while achievement of the continuity and patency of RPA on the MPA is the key technical difficulty. According to the experience of our center, the aortic arch repair should be completed first to facilitate systemic perfusion as early as possible. Ideally, the reconstructed aorta and the PA can both keep growing. However, as seen in our center together with the long-term follow-up in other literature (10), post-operative RPA stenosis is still the most common complication, owing to the difficulty in reconstruction and the compression due to a limited space next to the AAO. Bi et al. (1) found that many patients (58.6%, 17/29) suffered from post-operative RPA stenosis, and seven patients (41%, 7/17) underwent reinterventions, including five patients were performed surgical repair and three patients were performed balloon angioplasty. According to his binary logic regression analysis, anatomical morphology classification of berry syndrome, for example, the morphology of aortic artery, IAA type, APW type, patent ductus arteriosus type, original RPA site, and type of surgery, had nothing to do with post-operative complications and reintervention (1).

There are three types of surgical methods to re-establish the RPA continuity in patient with berry syndrome as reported by literatures (6). However, the optimal surgical treatment is still a controversial issue. One method is the intra-aortic baffle technique. According to the experience of Hu and his center (6), this method is suitable for all patients with type IIa APW, but only for the part of patients with type IIb APW depending on the distance between the APW and RPA. Hu and Mannelli et al. also described several cases of type IIb APW showing no complications after being treated by this method (11). While our second patient with IAA Type B and APW Type III in our center showed post-operative RPA stenosis, unfortunately after applying this method. Another method is the RPA cut-off and replantation technique. We applied this method in patient No. 1 and 3. RPA was anastomosed to MPA *in situ* in patient No. 1 and in front of the aorta with patch augmentation in patient No. 3 similarly to the LeCompte maneuver. This technique is the most frequently performed method to correct the isolated AROPA (12) and can be performed to treat any type of berry syndrome regardless the location of the RPA or the size of the APW. However, this technique is more difficult to perform as compared to the method of intra-aortic baffle, since it demanded the mastery of anatomical space for the chief surgeon to avoid complications like myocardial ischemia. Such complication had been reported by Duyen et al. (13) in a case of berry syndrome with direct implantation of RPA to MPA. The ischemia was caused by the distended and stretched RPA compressing the left coronary artery. Moreover, this method destroyed the original continuity between the RPA and MPA.

The third method is RPA arterioplasty with the aortic cuff technique. Abbruzzese (14) first reported this technique that an “*in situ*” reconstruction of the pulmonary continuity using native aortic tissue in a male infant weighing 3.800 kg. Then Park et al. (15) also reported another two cases using this method and with one case underwent percutaneous balloon angioplasty 1 year post-operatively. According to Hu et al. (6), the reoperation rate of this method was nearly 33.3%, which was much higher than the reoperation rate of the above-mentioned two methods. Moreover, this method was also quite similar to the double flap surgical technique described by Prifti et al., which employed native tissue from aortic and pulmonary flap to reduce the tension at the RPA–MPA anastomosis (16). Double flap surgical technique may be advantageous in offering extra autologous tissue length and enabling the placement of the newly created communicating tube anterior to the AAO. It can be a valuable technique and need a little modification for applying in the patient with berry syndrome. For now, considering the small sample size, short follow-up time, the variability of the location where the RPA arises from the posterior wall of the aorta, and the diversity of surgical techniques, it is difficult to determine which surgical method is better.

The phenotype of IAA type B combined with APW type III in our patient No. 2 was reported for the first time so far. But this was also an imperfect case for applying the intra-aortic baffle technique to repair APW and AORPA. Because of the huge defect and significant distance between the APW and RPA, the intra-aortic baffle technique may be suboptimal for the APW type III. Hemodynamically, the intra-aortic patch was pushed toward the PA owing to the pressure gradient between AAO and PA, which might cause RPA stenosis or even LPA stenosis and the potential problem of the left ventricular outflow tract. Binsalamah et al. (10) reported a case with type A of IAA and type III of APW showing a mild post-operative RPA stenosis by the technique similar to Park et al. (15). Ando et al. (9) also reported a similar case like Binsalamah but underwent two-stage surgical repair, and manifested that the maximal flow velocity of the aorta was 1.1 m/s and both RPA and LPA were 2.0 m/s. Thus, for the type III of APW, RPA arterioplasty with an aortic cuff technique seems to be more suitable, but further investigation and longer follow-up time are still needed.

In summary, berry syndrome can be safely corrected by one-stage surgical repair in experienced cardiac centers once diagnosed in the neonatal period. It requires not only an excellent team of cardiac surgeons, but also the collaboration of ultrasound, imaging, anesthesia, cardiopulmonary bypass, monitoring, and nursing teams. There was no operative death or late death in our cases, yielding a survival of 100% after repair, indicating the current era advances both in surgical and perioperative management strategies. Echocardiography combined with the contrast-enhanced CT scan can provide adequate diagnostic information. Compared to IAA type A, IAA type B in this syndrome shows a greater influence on systemic circulation. It is the first time that the combination of berry syndrome with type B of IAA and type III of APW has been reported so far. Early diagnosis and surgical treatment facilitate a good prognosis and offer satisfactory early and middle outcomes. But longer follow-up periods are still needed for the better characterization of long-term outcomes to determine that whether the growth and development are matching the needs.

## Data Availability Statement

The original contributions presented in the study are included in the article/supplementary material, further inquiries can be directed to the corresponding author.

## Ethics Statement

Written informed consent was obtained from the individual(s), and minor(s)' legal guardian/next of kin, for the publication of any potentially identifiable images or data included in this article.

## Author Contributions

X-CS: concept design, data collection, investigation, and manuscript writing, review and editing. J-BW: data collection and writing–review and editing. JY: ultrasound guidance and images analysis. X-HM: radiographic guidance and images analysis. Y-QP: drawing the schematic diagram of operation. L-YY: project guidance and supervision. J-GY: project guidance and supervision. All authors contributed to the article and approved the submitted version.

## Conflict of Interest

The authors declare that the research was conducted in the absence of any commercial or financial relationships that could be construed as a potential conflict of interest.

## Publisher's Note

All claims expressed in this article are solely those of the authors and do not necessarily represent those of their affiliated organizations, or those of the publisher, the editors and the reviewers. Any product that may be evaluated in this article, or claim that may be made by its manufacturer, is not guaranteed or endorsed by the publisher.
